# Nurses' knowledge about Berardinelli-Seip Congenital Lipodystrophy

**DOI:** 10.1371/journal.pone.0197784

**Published:** 2018-06-04

**Authors:** Verônica Kristina Cândido Dantas, Joice da Silva Soares, Lázaro Batista de Azevedo Medeiros, Aquiles Sales Craveiro Sarmento, Thaiza Teixeira Xavier Nobre, Fábia Barbosa de Andrade, Josivan Gomes de Lima, Julliane Tamara Araújo de Melo Campos

**Affiliations:** 1 Faculdade de Ciências da Saúde do Trairi, Universidade Federal do Rio Grande do Norte, Santa Cruz, RN, Brazil; 2 Laboratório de Biologia Molecular e Genômica, Departamento de Biologia Celular e Genética, Centro de Biociências, Universidade Federal do Rio Grande do Norte, Natal, RN, Brazil; 3 Departamento de Medicina Clínica, Hospital Universitário Onofre Lopes (HUOL)/UFRN, Natal, RN, Brazil; Chuo University, JAPAN

## Abstract

Berardinelli-Seip Congenital Lipodystrophy (BSCL) is a rare autosomal recessive disease characterized by the almost complete absence of adipose tissue. Due to a strong founder effect that resulted in a higher prevalence of BSCL in Rio Grande do Norte (*RN*), a state in northeastern Brazil, it has been essential that health professionals develop knowledge about this disease. Nurses are often the first point of contact with patients during health care assistance. The purpose of this study was to investigate the knowledge of these professionals about BSCL in two main hospitals in *RN* state. A questionnaire was applied to 199 nurses working in the *Hospital Regional Mariano Coelho*—*HRMC* (Regional Hospital Mariano Coelho), in *Currais Novos–RN*, and in the *Hospital Universitário Onofre Lopes*—*HUOL* (University Hospital Onofre Lopes), in *Natal–RN*. This study showed that most nursing professionals do not know about the disease, although they have already received patients with BSCL in those hospitals. The nurses from *HRMC* and *HUOL* lacked knowledge of BSCL and the healthcare of these patients requires immediate improvement. Significant efforts are required to close the gap between current and needed practice patterns.

## Introduction

Berardinelli-Seip Congenital Lipodystrophies (BSCLs) are autosomal recessive disorders characterized by a generalized loss of adipose tissue from birth, hypertriglyceridemia, hyperinsulinism, glucose intolerance, hepatic steatosis, diabetes *mellitus*, prominent musculature, hypertrophic cardiomyopathy, bone cysts, umbilical protrusion, acanthosis nigricans, and other clinical features [1–6].

The prevalence rate of BSCL in different countries has been estimated in the literature. In the US and Norway, the prevalence of this disease is 1 in 10 million and 1 in 1 million people, respectively [3,7]. However, in Lebanon, Portugal, and Oman, the prevalence rates are 1 in 200,000, 1 in 500,000, and 1 in 25,000 people, respectively [7]. Despite the absence of a regular registration system for BSCL at the national level, our research found that *RN* presents a high BSCL prevalence (3 in 100,000), mainly in *Seridó* territory due to consanguineous marriages [8]. Clinical and laboratory data for BSCL patients from *RN* were reported by Lima et al [9].

As an extremely rare lipodystrophy, BSCL is almost unknown by the health professionals. However, the great prevalence in *RN*, compared with the rest of the world, raises the necessity of specialized knowledge by those professionals. Besides, financial and human resources are still necessary to manage patients with lipodystrophies in *RN*. Thus, the *ASPOSBERN* (*Associação de Pais e Pessoas com a Síndrome de Berardinelli do Estado do Rio Grande do Norte—*Association of Parents and People with Berardinelli Syndrome of Rio Grande do Norte) Association was founded as a non-profit organization aiming to improve the quality of life of BSCL patients and their families, by performing an important role in the management of BSCL patients diagnosed by qualified physicians [8,9].

Brazilian nursing teams are essentially comprised of three occupational groups, according to the nurse training levels: nurses with higher education (referred to here as ‘nurses’), ‘nursing technician’ with secondary education, and ‘nursing aid’ with basic education. In Brazil, nurse technicians and nurses represent most of the nursing workforce. These workers are mainly responsible for activities that involve direct contact with patients. However, these two different nursing categories comprise different responsibilities and roles: Nurses are in charge of teaching, supervision, and management, while technical and auxiliary staff carry out most care activities [10].

Nursing care for patients with BSCL is essential from birth and requires specialized knowledge to ensure the safety of the patients and their families. The care and surveillance of BSCL patients by nurses should include blood pressure monitoring; periodic screening for glycosuria and proteinuria; glucose, glycated hemoglobin, triglyceride, insulin, leptin, alanine aminotransferase (ALT), and aspartate aminotransferase (AST) levels; bone age, signs of precocious puberty, and genetic counseling may also be taken into account [11–13].

Since nurses are the largest care workforce for BSCL patients in hospitals, a valid and reliable instrument to evaluate their knowledge, skills, and attitudes is essential to help the Brazilian Health System promote strategies and genetic counseling for families, and improving the management of individual abnormalities of this type of lipodystrophy is a priority. For this purpose, this study aimed to evaluate the knowledge, skills, and practice related to BSCL of nurses at two hospitals located in Rio Grande do Norte state, Northeast Brazil.

## Materials and methods

### Study design and data collection

**Fig 1:**
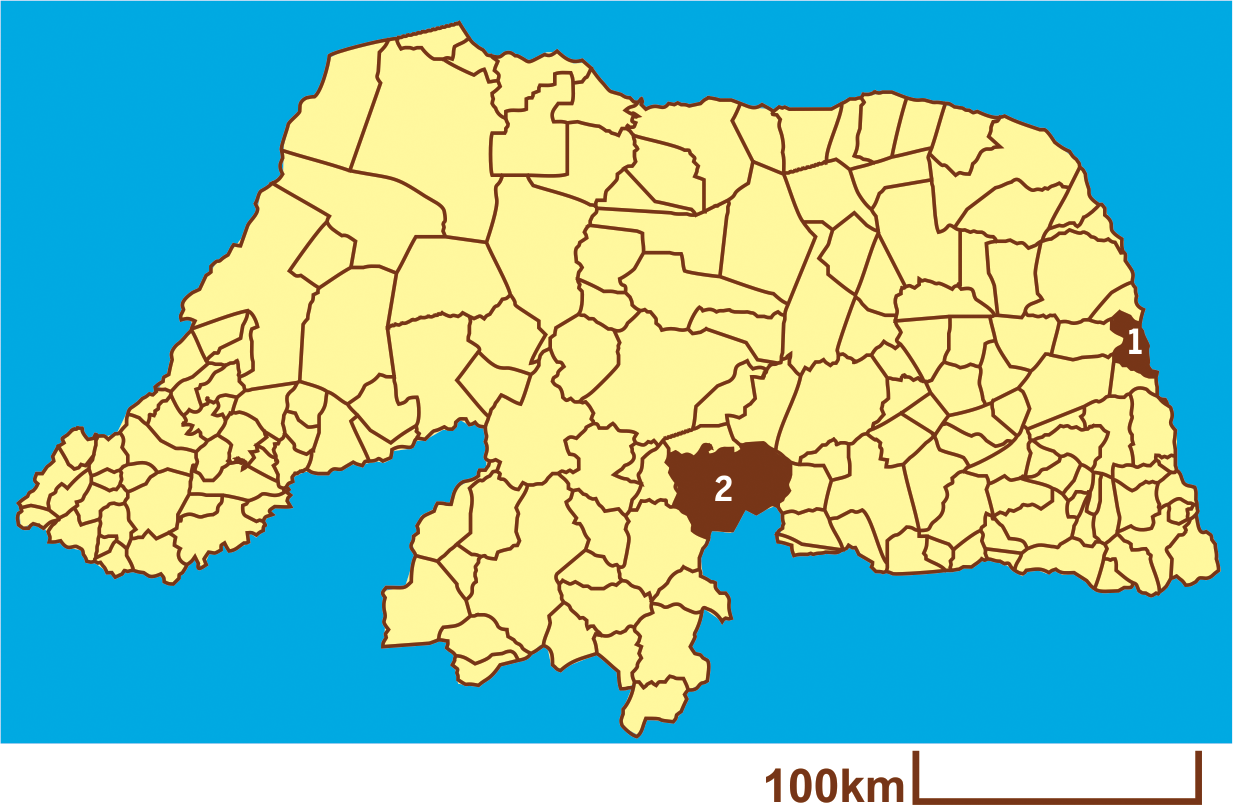
Map of the *Rio Grande do Norte* state. Geolocalization of *Natal* (1) and *Currais Novos* (2), where *HUOL* and *HRMC* hospitals are located, respectively. The map was created using the TabWin Program (DATASUS/*Ministério da Saúde*–Brazilian Ministry of Health).

In this cross-sectional study, 199 nurses were randomly selected from two community hospitals from Rio Grande do Norte state, Northeast Brazil. The *Hospital Regional Mariano Coelho*—*HRMC* (Regional Hospital Mariano Coelho) and the *Hospital Universitário Onofre Lopes*—*HUOL* (University Hospital Onofre Lopes) are located in the cities of *Currais Novos* and in *Natal*, respectively (Fig 1).

The study data were collected from January to August 2015. All participants were requested to complete a questionnaire aiming to evaluate their knowledge, attitude, and skills related to BSCL. All nurses were verbally informed about the study by the principal investigator, and signed a written consent before participation.

An appropriate instrument which could measure the knowledge of BSCL by nurse professionals was developed specifically for this study and was divided into two parts: The first included general information on the participants (gender, age, occupation, educational level, job titles, specialty, clinical experience time); and the second concerned the participants’ knowledge about BSCL (S1 and S2 Data Sets). Then, this questionnaire was pre-tested and an expert consultation verified the validity of the content.

### Statistical analysis

The variables of this study were age, sex, level of education, and clinical experience time. The data were analyzed using Statistical Package for Social Sciences software (SPSS Inc., Chicago, USA), version 20.0. The number of participants was expressed in absolute number and percentage. Data were presented as number (%) for categorical variables. Continuous and normally distributed variables were expressed as mean ±SD. The Fisher’s exact test was performed to verify the differences between both hospitals and between nurses and technicians. The level of significance was set at 0.05.

### Ethical statement

The consent procedure and research were approved by the Ethics and Research Committee of *Faculdade de Ciências da Saúde do Trairi—FACISA* (Faculty of Health Sciences of Trairi) of *Universidade Federal do Rio Grande do Norte—UFRN* (Federal University of Rio Grande do Norte) with study number 36182614.1.0000.5568. The data did not contain any personal identifiers. The confidentiality of all information was preserved.

## Results

### General information of participants

199 questionnaires were included in this research: 75 completed by nurses and 124 by nursing technicians from both hospitals. Of the 199 participants, there were 111 (56%) females, aged from 20 to 59 years old, with a mean of 37.8 ± 9.8 years. When we analyzed the data of the two hospitals separately, 155 and 44 participants were from *HUOL* and *HRMC* (nurses and technicians), respectively. For *HUOL*, there were 64 nurses (38.6±9.9 years old, 60 females) and 91 technicians (33.7±7.8 years old, 13 females). For *HRMC*, there were 11 nurses (36.5 ± 8.2 years old, 9 females) and 33 technicians (47.4±7.9 years old, 29 females) (Table 1). The mean age of nurses at *HUOL* and *HRMC* was not different (p = 0.5). However, technicians from *HRMC* were older than those from *HUOL* (p < 0.0001). On the other hand, when we verified the mean age of participants from *HUOL*, we observed that the nurses were older than technicians (p = 0.0010), while for *HRMC* the data showed that technicians were older than nurses (p < 0.0004). In fact, for *HUOL*, the age group ≥ 50 years was 8% and 3% for nurses and technicians, respectively, whereas for *HRMC* we found that 18% and 48% of nurses and technicians, respectively, were in the age group of ≥ 50 years (Table 1). Taken together, these data reflect the similarity in the age pattern between nurses of *HUOL* and *HRMC* but not between technicians, and a contrasting age pattern between nurses and technicians who work in the same hospital.

**Table 1:** Personal status between nurses and technicians from *HUOL* (N = 155) and *HRMC* (N = 44).

N (%)	Nurses *HUOL* (N = 64)	Nurses *HRMC* (N = 11)	Nurses Total (N = 75)	Technicians *HUOL* (N = 91)	Technicians *HRMC* (N = 33)	Technicians Total (N = 124)	Total number (N = 199)
Gender							
Male	4 (6.0)	2 (18)	6 (8.0)	78 (87)	4 (12)	82 (66)	88 (44)
Female	60 (94)	9 (81)	69 (92)	13 (13)	29 (88)	42 (34)	111 (56)
Age Group (Mean ± SD)	38.6±9.9	36.5±8.2	38.35±9.7	33.7±7.8	47.4±7.9	37.57±10.0	37.86±9.8
≤35 Years	26 (40)	6 (55)	32 (43)	57 (63)	3 (9)	60 (49)	92 (47)
36–49 Years	28 (44)	3 (27)	31 (41)	27 (30)	14 (42)	41 (33)	72 (36)
≥50 Years	5 (8.0)	2 (18)	7 (9.0)	3 (3.0)	16 (48)	19 (15)	26 (13)
Unknown	5 (8.0)	0 (0.0)	5 (7.0)	4 (4.0)	0 (0.0)	4 (3.0)	9 (4.0)
Education Level							
Technical secondary school graduates or below	0 (0.0)	0 (0.0)	0 (0.0)	91 (100)	33 (100)	124 (100)	124 (62)
Specialization	48 (75)	08 (72)	56 (75)	0 (0.0)	0 (0.0)	0 (0.0)	56 (28)
Master or higher degree	15 (23)	0 (0.0)	15 (20)	0 (0.0)	0 (0.0)	0 (0.0)	15 (7.0)
Unknown	01 (2.0)	03 (28)	04 (5.0)	0 (0.0)	0 (0.0)	0 (0.0)	4 (3.0)
Clinical experience time (years)							
0–10	26 (41)	7 (64)	33 (44)	39 (43)	2 (6.0)	41 (33)	74 (37)
11–20	23 (36)	1 (9.0)	24 (32)	9 (10)	5 (16)	14 (11)	38 (20)
21–30	6 (9.0)	1 (9.0)	7 (9.0)	0 (0.0)	12 (36)	12 (10)	19 (10)
>30	4 (6.0)	0 (0.0)	4 (6.0)	0 (0.0)	1 (3.0)	1 (1.0)	5 (2.0)
Unknown	5 (8.0)	2 (18)	7 (9.0)	43 (47)	13 (39)	56 (45.0)	63 (31)

Concerning the education level, of the 199 nurses who participated in this study, 28% had specialization and only 7% had a master or higher degree, resulting in 35% professionals with a postgraduate degree (Table 1). When we analyzed the clinical experience time, 41% of the nurses from *HUOL* and 64% of the nurses from *HRMC* had between 0 to 10 years of clinical experience. However, when we analyzed this status for technicians at the respective hospitals, the results showed that 43% and 6% were professionals with 0 to 10 years of clinical experience. Additionally, we found that 36% of the nurses from *HUOL* and 9% of the nurses from *HRMC* had between 11 to 20 years of clinical experience. For technicians at the respective hospitals, 10% and 16% had between 11 to 20 years of clinical experience. 36% of the technicians at *HRMC* and 43% of those at *HUOL* had between 21 to 30 years of clinical experience. For more than 30 years of experience, *HUOL* presented 6% of nurses, but no technicians. At *HRMC*, no nurses and only 3% of technicians presented more than 30 years of experience. Together, these data suggest that nurses from *HUOL* had higher clinical experience time than those from *HRMC*, while technicians from *HRMC* had more clinical experience time than those from *HUOL*.

### BSCL knowledge

When nurses and technicians answered questions about BSCL morpho-physiological features, the data showed opposite results between *HUOL* and *HRMC* (Tables 2 and 3). While 64% of the nurses and 79% of the technicians from *HRMC* had knowledge about the morpho-physiological aspects of BSCL (p = 0.4247), only 36% of the nurses and 10% of the technicians from *HUOL* affirmed to recognize these features (p = 0.0001). When we verified their knowledge about the genetic causes of BSCL, 36% of the nurses and 21% of the technicians from *HRMC* were knowledgeable of such causes of BSCL (p = 0.4247). For *HUOL*, 11% of the nurses and 4.0% of the technicians had any information about BSCL genetic causes (p = 0.2019). Afterward, they were asked about their ability to recognize a BSCL patient. While 64% of the nurses and 70% of the technicians from *HRMC* informed that they could recognize a patient with BSCL (p = 0.7222), 19% of the nurses and 4% of the technicians from *HUOL* affirmed that they had the ability to recognize these patients (p = 0.0060). Subsequently, when they were asked if they had assisted the treatment of a BSCL patient in their hospital, 45% of the nurses and 42% of the technicians from *HRMC* answered positively (p = 0.1000), while only 19% of the nurses and 2% of the technicians from *HUOL* had assisted the treatment of a BSCL patient (p = 0.0009).

**Table 2:** The knowledge of nurses and technicians from *HRMC* about Berardinelli-Seip Congenital Lipodystrophy (BSCL).

Yes—N (%) No—N (%)		Nurses *HRMC* (N = 11)	Technicians *HRMC* (N = 33)	Total HRMC (N = 44)	P	OR	95% CI
Q1. Have knowledge about the morpho-physiological aspects of BSCL.		7 (64) 4 (36)	26 (79) 7 (21)	33 (75) 11 (25)	0.4247	0.4712	(0.1155, 1.793)
Q2. Have knowledge about the genetic causes of BSCL.		4 (36) 7 (64)	7 (21) 26 (79)	11 (25) 33 (75)	0.4247	2.122	(0.5576, 8.654)
Q3. Can recognize a BSCL patient.		7 (64) 4 (36)	23 (70) 10 (30)	30 (68) 14 (32)	0.7222	0.7609	(0.2061, 2.755)
Q4. Had any BSCL patient in your hospital.		5 (45) 6 (55)	14 (42) 19 (57)	19 (43) 25 (57)	0.1000	1.131	(0.2931, 3.898)
Q5. Can explain to the parents of a new baby with BSCL the morpho-physiological and genetic causes of this disease.		2 (18) 9 (82)	2 (06) 31 (94)	4 (9) 40 (91)	0.2565	3.444	(0.4713, 23.55)
Q6. Can properly source the health care for a BSCL patient.		3 (27) 8 (73)	8 (24) 25 (76)	11 (25) 30 (75)	1.000	1.172	0.2818, 4.799)
Q7. Have heard about *ASPOSBERN*.		6 (55) 5 (45)	19 (58) 14 (42)	25 (57) 19 (43)	1.000	0.8842	(0.2565, 3.411)

OR: Odds Ratio.

95% CI: 95% Confidence Interval.

All statistical tests were performed using p-value < 0.05 as the level of significance.

**Table 3:** The knowledge of nurses and technicians from *HUOL* about Berardinelli-Seip Congenital Lipodystrophy (BSCL).

Yes—N (%) No—N (%)	Nurses *HUOL* (N = 64)		Technicians *HUOL* (N = 91)	Total *HUOL* (N = 155)	P	OR	95% CI
Q1. Have knowledge about the morpho-physiological aspects of BSCL.	23 (36) 41 (64)		9 (10) 82 (90)	32 (21.0) 123 (79.0)	0.0001	5.111	(2.146, 11.94)
Q2. Have knowledge about the genetic causes of BSCL.	7 (11) 57 (89)		4 (4.0) 87 (96)	11 (7.0) 144 (93)	0.2019	2.671	(0.7925, 8.404)
Q3. Can recognize a BSCL patient.	12 (19) 52 (81)		4 (4.0) 87 (96)	16 (11.3) 139 (89.7)	0.0060	5.019	(1.675, 14.68)
Q4. Had any BSCL patient in your hospital.	12 (19) 52 (81)		2 (2.0) 89 (98)	14 (10) 141 (90)	0.0009	10.27	(2.389, 46.91)
Q5. Can explain to the parents of a new baby with BSCL the morpho-physiological and genetic causes of this disease.	5 (7.8) 59 (92.2)		1 (1.0) 90 (99)	6 (4.0) 149 (96.0)	0.0822	7.62	(0.992, 90.71)
Q6. Can properly source the health care for a BSCL patient.	5 (7.8) 59 (92.2)		1 (1.0) 90 (99)	6 (4.0) 149 (96.0)	0.0822	7.62	(0.992, 90.71)
Q7. Have heard about *ASPOSBERN*.	3 (5.0) 61 (95)		4 (4.4) 87 (95.6)	7 (4.5) 148 (95.5)	1.000	1.07	(0.262, 4.1)

OR: Odds Ratio.

95% CI: 95% Confidence Interval.

All statistical tests were performed using p-value < 0.05 as the level of significance.

When we asked about their skills to better inform a family of a recently born BSCL patient regarding the morpho-physiological features and genetic causes, 18% of the nurses and 6% of the technicians from *HRMC* answered positively (p = 0.2565). On the other hand, only 7.8% of the nurses and 1% of the technicians from *HUOL* affirmed the same (p = 0.0822). With regard to providing health care to BSCL patients, 27% of the nurses and 24% of the technicians from *HRMC* affirmed that they had the necessary knowledge and skills to offer BSCL patients the proper health care (p = 1.000). On the other hand, only 7.8% of the nurses and 1% of the technicians from *HUOL* affirmed that they had the ability to provide BSCL patients the proper health care (p = 0.0822). Finally, when they were asked about their knowledge regarding the *ASPOSBERN* association, 55% of the nurses and 58% of the technicians from *HRMC* affirmed their having knowledge about the activities performed by this association (p = 1.000), while only 5% of the nurses and 4.4% of the technicians from *HUOL* had the same knowledge (p = 1.000). All of these data are summarized in Tables 2 and 3. When the data were analyzed together for each hospital, we observed that *HRMC* nurses and technicians had more knowledge for almost all questions compared to their counterparts at *HUOL* (Table 4).

**Table 4:** The knowledge of nurse professionals from HUOL and HRMC about Berardinelli-Seip Congenital Lipodystrophy (BSCL).

Yes—N (%) No—N (%)	HUOL (N = 155)	HRMC (N = 44)	Total (N = 199)	P	OR	95% CI
Q1. Have knowledge about the morpho-physiological aspects of BSCL.	32 (20.6) 123 (79.4)	33 (75.0) 11 (25.0)	65 (32.7) 134 (67.3)	< 0.0001	0.08672	(0.04166, 0.1927)
Q2. Have knowledge about the genetic causes of BSCL.	11 (7.1) 144 (92.9)	11 (25.0) 33 (75.0)	22 (11.1) 177 (88.9)	0.0021	0.2292	(0.09209, 0.5731)
Q3. Can recognize a BSCL patient.	16 (10.3) 139 (89.7)	30 (68.2) 14 (31.8)	46 (23.1) 153 (76.9)	< 0.0001	0.05372	(0.02529, 0.1206)
Q4. Had any BSCL patient in your hospital.	14 (9.0) 141 (91.0)	19 (43.2) 25 (56.8)	33 (15.6) 166 (83.4)	< 0.0001	0.1306	(0.05775, 0.302)
Q5. Can explain to the parents of a new baby with BSCL the morpho-physiological and genetic causes of this disease.	06 (3.9) 149 (96.1)	04 (9.0) 40 (90.0)	10 (5.0) 189 (95.0)	0.2325	0.4027	(0.1073, 1.321)
Q6. Can properly source the health care for a BSCL patient.	06 (3.9) 149 (96.1)	11 (25.0) 33 (75.0)	17(8.5) 182 (97.5)	< 0.0001	0.1208	(0.04152, 0.3698)
Q7. Have heard about *ASPOSBERN*.	07 (4.5) 148 (95.5)	25 (57) 19 (43)	32 (16) 167 (84)	< 0.0001	0.3595	(0.01485, 0.09315)

OR: Odds Ratio.

95% CI: 95% Confidence Interval.

All statistical tests were performed using p-value < 0.05 as the level of significance.

After responding to all of the questions, all of the participants were invited to discuss BSCL morphology, physiology, genetic causes, treatment, and the importance of multi-professional assistance for these patients. However, 33% (n = 50) and 50% (n = 22) of nursing professionals from *HUOL* and *HRMC*, respectively, participated in those discussions.

## Discussion

In the current study, the knowledge of nurses and technicians in two hospitals from *Rio Grande do Norte* state (*RN*), Brazil, regarding BSCL was determined. Although we have previously estimated a high BSCL prevalence rate of 3.23 per 100,000 people in *RN* [8], we found that the nurses and technicians from *HUOL*, located in the capital city of *RN*, lacked knowledge concerning the BSCL morpho-physiological and genetic causes. It should be noted that *HUOL* is the main hospital in *RN* that promotes the health care of BSCL patients and serves as the major reference for qualified physicians on the treatment of BSCL [9]. On the other hand, nurses and technicians from *HRMC*, in *Currais Novos*, located in *Seridó* territory in *RN*, presented more knowledge concerning BSCL than those from *HUOL*: 75% had knowledge about the morpho-physiological aspects; they could recognize a patient with BSCL; almost 45% had participated in a patient treatment; and 57% knew about the *ASPOSBERN* Association (Table 4). This is the first study to screen the knowledge of nurses from Rio Grande do Norte state (*RN*), Brazil, regarding BSCL.

Although the responsibilities of nurses and nursing technicians are determined according to their education level, we found a greater lack of BSCL knowledge between these two categories only at *HUOL*, the main hospital in *RN* that promotes the regular surveillance and therapeutic management of BSCL patients. Since *HRMC* and *ASPOSBERN* are both located in *Currais Novos* city, we believe that the *ASPOSBERN* activities could explain the higher knowledge of both nurses and technicians from that location. Another explanation is that *HRMC* technicians were older than those at *HUOL* (47.7 ± 7.9 vs 33.7 ± 7.8) and 48% of them were ≥ 50 years. Although technicians presented only a secondary education, these age data could also explain why this *HRMC* category presented higher knowledge concerning BSCL, since they lived in the *Seridó* territory, which has presented many BSCL cases since the end of the 20^th^ century. We believe that the higher level of knowledge found in *Currais Novos* municipality was due to both the elevated age of *HRMC* technicians and *ASPOSBERN* actions in that *RN* territory. Taken together, these findings highlight the urgent requirement to develop efforts directed at offering education and training to ensure that health care providers have suitable knowledge and skills to properly treat BSCL patients mainly at *HUOL*.

This lipodystrophy was initially described in 1954 by a Brazilian doctor, Waldemar Berardinelli [1], and in 1959 by a Norwegian doctor, Martin Seip [14]. *RN* patients with this type of lipodystrophy present a phenotype very well characterized: acromegaloid facial appearance, atrophic cheeks, prognathism, prominent musculature, umbilical protrusion, acanthosis nigricans, phlebomegaly, and others [6,9,15–18]. These morphological characteristics can be easily identified and in the last years an increase in the number of diagnosed patients has been observed [6,8,9,15].

**Fig 2:**
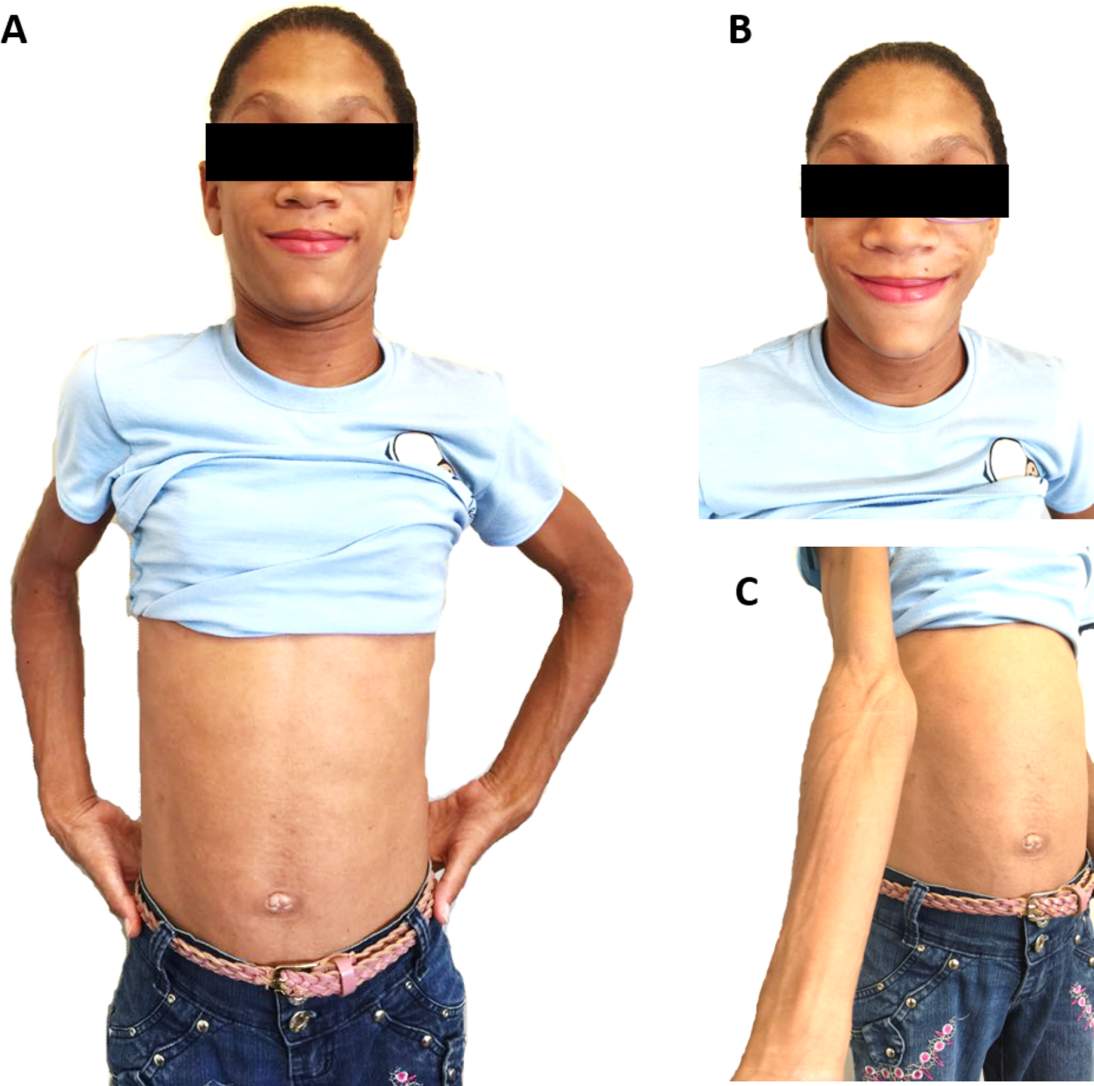
Physical appearance of type 1 BSCL patient from *RN*. (A) Anterior view of a 24-year-old Brazilian female with type 1 BSCL due to *A712T* mutation in the *AGPAT2* gene. (B) Anterior view of face showing acromegaloid facies, atrophic cheeks, and prognathism. (C) Anterior view of right arm and abdomen showing phlebomegaly and umbilical protusion, respectively, as previously described by Lima and co-workers [6,9].

**Fig 3:**
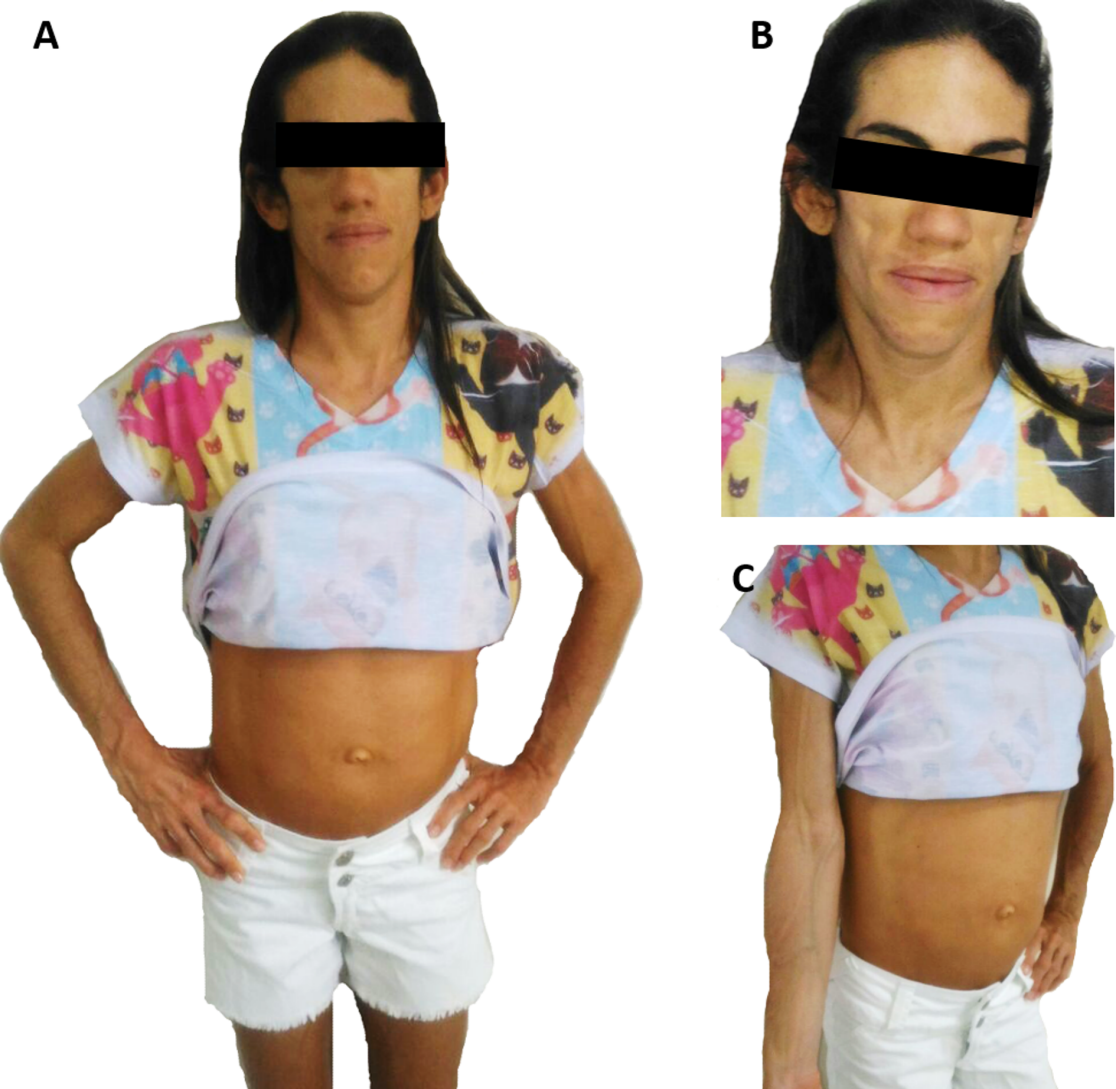
Physical appearance of type 2 BSCL patient from *RN*. (A) A view of a 33-year-old Brazilian female with type 2 BSCL due to *325dupA* mutation in *BSCL2* gene. (B) Anterior view of face showing acromegaloid facies, atrophic cheeks, and prognathism. (C) Anterior view of right arm and abdomen showing phlebomegaly and umbilical protusion, respectively, as previously described by Lima and co-workers [6,9].

**Fig 4:**
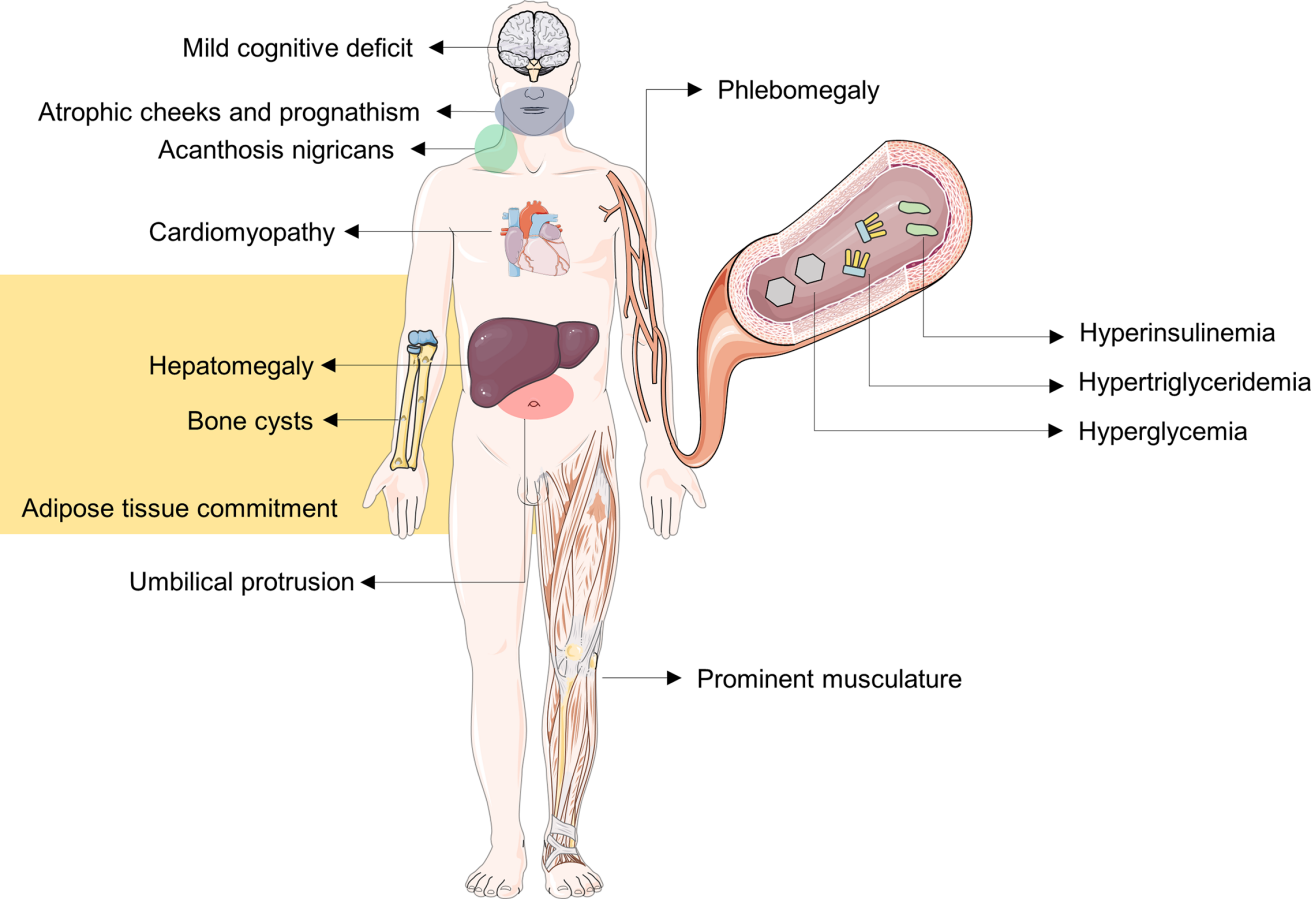
A schematic overview of the main morphological features of type 1 and type 2 BSCL patients. Acanthosis nigricans, acromegaloid facies, atrophic cheeks, prognathism and phlebomegaly, and metabolic comorbidities are shown, as previously described by Lima and co-workers [9].

Depending on the case, the diagnosis of BSCL can be established by associating clinical characteristics with biochemical and/or genetic exams. The characteristics that determine the majority of the criteria are acromegaloid facial appearance, hepatomegaly, high concentration of triglycerides (> 150 mg/dL), and insulin resistance. The characteristics of the minor criteria are hypertrophic cardiomyopathy, mild psychomotor retardation or moderate cognitive deficit, hirsutism, and puberty in pre-school girls. However, due to its rarity, most clinical physicians and health care providers are not familiar with its diagnosis and management [12]. Figs 2 and 3 show the physical appearance of type 1 and type 2 BSCL patients from *RN*. Also provided is a schematic overview (Fig 4) highlighting the main morphological features found in BSCL from *RN* to emphasize that nurses must be aware of those features to help the recognition of those patients, which can have great implications for health care based on body images.

According to data from the literature [9,12,15], the BSCL surveillance needs to be achieved periodically during the year to monitor hepatic complications and the clinical parameters of occurrence/progression of diabetes mellitus. Furthermore, since BSCL is a metabolic disease that affects several systems, a multidisciplinary team composed of physicians, nurses, nutritionists and dietitians, physical therapists, geneticists, and others is required to ensure the suitable surveillance.

Nutritional advice can also be employed by nurses to help families. These professionals can inform the requirement to consult a dietitian to acquire a specialized diet, with a focus on balanced macronutrient composition comprising 50–60% carbohydrate, 20–30% fat, and 10–20% protein, as well as including the adequacy of micronutrient intakes. To reduce hypertriglyceridemia, the nurses can suggest the intake of medium chain triglyceride-based formulas in infants and older individuals. Cis-mono-unsaturated fats and long chain omega-3 fatty acids should be the main form of fat intake. This is critical conduct that needs to be offered after birth since diet is a basis to prevent or ameliorate the metabolic comorbidities of BSCL [19–24]. According to the World Health Organization (WHO) and *Instituto Brasileiro de Geografia e Estatística*—*IBGE* (Brazilian Institute of Geography and Statistics) [25,26], more than 50% of Brazilian adults are overweight and 20% are obese. Additionaly, it is important to note that *RN* is one of the Brazilian states with a higher number of overweight and obese individuals [27]. Since *Seridó* territory in *RN* is known for its typical fatty food, such as coalho’s cheese, animal by-products, sugar cookies, and others, BSCL individuals need to avoid this type of food and the nurses can promote actions to ensure the nutrition and hydration requests of BSCL patients to improve their quality of life.

The importance of knowledge about the genetic causes of BSCL is crucial to predict the future course of the disease since specific symptoms can be associated with the type of mutation in certain genes. Mutations in genes *AGPAT2*, *BSCL2*, *CAV1*, and *CAVIN1* give rise to 4 subtypes of BSCL: type 1, 2, 3, and 4, respectively [5]. In *RN* state from Brazil, only type 1 and type 2 of BSCL were described [8]. Type 1 BSCL occurs due to mutations in the gene *AGPAT2* that encode 1-acylglycerol-3-phosphate O-acyltransferase 2 (1-AGPAT 2), while type 2 BSCL is caused by mutations in the gene *BSCL2* that encode seipin [28,29].

Concerning genetic consequences, mutations in the *AGPAT2* gene are associated with the occurrence of bone cysts [12,30], while mutations in the *BSCL2* gene are associated with cognitive deficits and a higher incidence of premature death [8,31]. According to Brown et al., genetic diagnosis is crucial for prenatal diagnosis and genetic counseling [12]. Thus, the genetic knowledge of BSCL should help the healthcare providers to offer better surveillance and to direct the correct choice of specific therapeutic tools. Here, we found that the nurses from *HUOL* and *HRMC* lacked knowledge about the genetic causes of BSCL and this finding was more significant for *HUOL*.

Nursing professionals can also offer some advice on the importance of the practical physical exercises to ameliorate the metabolic complications of BSCL patients. Since the lack of physical exercise is a central mechanism in the development of diabetes mellitus [32–34], they should be encouraged to exercise, taking into account the comorbidities previously described, such as cardiomyopathies, bone cysts, hepatosplenomegaly, and others [9,15–18,35]. For patients presenting bone cysts and hepatosplenomegaly, contact sports are not appropriate. Protective shoes can be suggested in the case of patients with fractures in bone cysts. On the other hand, patients who have cardiomyopathy need to be evaluated before starting an exercise regimen [12]. In this context, the morphological, metabolic, and genetic knowledge of this generalized lipodystrophy can help to offer better exercise advice for each patient.

Even though the nursing professionals do not have the competence to supply the clinical diagnosis of any disease, it is extremely important that these professionals present the proper knowledge and skills to help the BSCL families concerning the health care required to the management of the individual abnormalities of this lipodystrophy.

This study has some limitations. Firstly, the survey was conducted in both urban and rural hospitals, but we need to improve the number of participants from HUOL, since after 2015 the number of nurse professionals increased in this hospital. Secondly, not all knowledge of BSCL management was assessed in the test questionnaire. Despite these limitations, a reasonably high response rate of 55% and 89% at *HUOL* and *HRMC*, respectively, was attained; therefore, the results represent nurses’ current knowledge of BSCL and healthcare for BSCL patients.

## Conclusions

Taken together, our data emphasize that nurses from *HRMC* and *HUOL* clearly lacked knowledge regarding Berardinelli-Seip Congenital Lipodystrophy. However, this finding was more pronounced for *HUOL* than for *HRMC*. Our data demonstrate that the healthcare of BSCL patients urgently needs to be improved and significant efforts are required to close the gap between current and desirable nursing practice patterns.

## Supporting information

S1 Data Set Questionnaire in Portuguese. (DOCX)

S2 Data Set Questionnaire in English. (DOCX)

S3 Data Set Individual participant data. (XLSX)
