# Supplementary material for: Nurses' knowledge about Berardinelli-Seip Congenital Lipodystrophy
Source: PLoS One. 2018 Jun 4;13(6):e0197784. doi: 10.1371/journal.pone.0197784 (PMC5986131; doi:10.1371/journal.pone.0197784)
Supplement: S1 Data Set — (DOCX) [file pone.0197784.s001.docx]

**
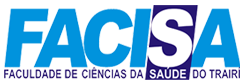
**

**PROJETO DE PESQUISA:**

**ESTUDO SOBRE O CONHECIMENTO DOS ENFERMEIROS ACERCA DA SÍNDROME DE BERARDINELLI-SEIP (BSCL) EM DOIS HOSPITAIS NO ESTADO DO RIO GRANDE DO NORTE**

Instruções:

O questionário a seguir contém 7 perguntas específicas sobre o conhecimento geral acerca da Síndrome de Berardinelli-Seip (Da questão 11 a 17). Você deverá escolher apenas uma resposta de cada pergunta, colocando um (x) naquela que julgar adequada.

Não há resposta certa ou errada. Você deverá marcar aquela que julgar mais adequada para seu caso ou situação.

Obrigada pela sua participação!

**Dados Pessoais**

1. Nome: ______________________________________________________________
2. nº de identificação ­­­­_______ nº de telefone para contato _______________________
3. Data da entrevista: ___/___/______ Data de Nascimento: ___/___/______
4. Idade: _____ anos
5. Sexo: ( ) M ( ) F.
6. E-mail: ______________________________________________________________

**Dados de Atuação/Qualificação Profissional**

1. Hospital em que trabalha: _______________________________________________
2. Quanto tempo você trabalha como enfermeiro? ______________________________
3. Formação: Técnico ( ) ou Graduado ( )
4. Pós-Graduação: ( ) Sim ( ) Não

Especialização ( )
Mestrado ( )

Doutorado ( )

Em qual área: ________________________________________________________

**Dados sobre o Conhecimento dos Enfermeiros acerca da Síndrome de Berardinelli-Seip**

1. Você conhece as causas morfofisiológicas da Síndrome de Berardinelli-Seip? ( ) Sim ( ) Não
2. Você conhece quais as causas genéticas da Síndrome de Berardinelli-Seip? ( ) Sim ( ) Não
3. Você saberia identificar um portador da Síndrome de Berardinelli-Seip que chega ao Serviço de Saúde? ( ) Sim ( ) Não
4. Você já teve algum paciente com a Síndrome de Berardinelli-Seip no seu Hospital?

( ) Sim ( ) Não

1. Ao nascer um paciente com a Síndrome de Berardinelli-Seip em seu serviço de saúde, você saberia explicar aos pais as causas morfofisiológicas e genéticas da Síndrome de Berardinelli-Seip? ( ) Sim ( ) Não
2. Você saberia repassar para as pessoas com a Berardinelli-Seip os cuidados que os mesmos deverão ter com a sua saúde? ( ) Sim ( ) Não

17. Você já ouviu falar da Associação dos Pais e Pessoas com a Síndrome de Berardinelli do Estado do Rio Grande do Norte (ASPOSBERN), cediada na cidade de Currais Novos? ( ) Sim ( ) Não
