# Supplementary material for: Nurses' knowledge about Berardinelli-Seip Congenital Lipodystrophy
Source: PLoS One. 2018 Jun 4;13(6):e0197784. doi: 10.1371/journal.pone.0197784 (PMC5986131; doi:10.1371/journal.pone.0197784)
Supplement: S2 Data Set — (DOCX) [file pone.0197784.s002.docx]

**
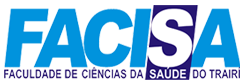
**

**RESEARCH PROJECT:**

**STUDY ABOUT NURSING KNOWLEDGE CONCERNING BERARDINELLI-SEIP CONGENITAL LIPODYSTROPHY (BSCL) IN TWO HOSPITALS FROM RIO GRANDE DO NORTE STATE**

Instructions:

The questionnaire contains 7 specific questions about the general knowledge concerning Berardinelli-Seip lipodystrophy (from 11 to 17). You can choose only 1 answer for each question matching a (x).

There is no a true or false answer. You must to match the better choice for you.

Thank you for your participation!!!

**Personal data**

1. Name: ______________________________________________________________
2. Register number ­­­­__________________ Phone number _______________________
3. Data of the questionnaire application: ___/___/___ Birth date: ___/___/___
4. Age: _____ years
5. Sex: ( ) M ( ) F.
6. E-mail: ______________________________________________________________

**Profissional data**

1. Name of the Hospital that you work: _______________________________________
2. How many time do you work in that Hospital? _______________________________
3. How your education level: Nursing technician ( ) or nurse ( )
4. Do you have a graduation: ( ) Yes ( ) No

Specialization ( )
Master ( )

PhD ( )

In which area? ________________________________________________________

**Data about the knowledge concerning Berardinelli-Seip Congenital Lipodystrophy**

1. Do you know the morpho-physiological features of Berardinelli-Seip Congenital Lipodystrophy? ( ) Yes ( ) No
2. Do you know the genetic causes of Berardinelli-Seip Congenital Lipodystrophy?

( ) Yes ( ) No

1. Could you recognize a patient with Berardinelli-Seip Congenital Lipodystrophy?

( ) Yes ( ) No

1. Do you had any BSCL patient in your hospital? ( ) Yes ( ) No
2. Can you explain to the parents of a new baby with BSCL the morpho-physiological and genetic causes of this disease? ( ) Yes ( ) No
3. Can you properly source the health care for a BSCL patient? ( ) Yes ( ) No
4. Do you have heard about *ASPOSBERN (Association of Parents and People with Berardinelli Syndrome of Rio Grande do Norte)*, located at Currais Novos city?

( ) Yes ( ) No
